# Correction: Specialized ribosomes and specific ribosomal protein paralogs control translation of mitochondrial proteins

**DOI:** 10.1083/JCB.20170605901292018c

**Published:** 2018-03-05

**Authors:** Nadav Segev, Jeffrey E. Gerst

Vol. 217, No. 1, January 2, 2018. 10.1083/jcb.201706059.

The first version of this article published online included errors in Fig. 3 d that occurred during production. The x axis of the two graphs in panel d had labels for “*rpl2aΔ*” and “*rpl2bΔ*” that should have been “*rpl1aΔ*” and “*rpl1bΔ*,” respectively.

**Figure 3.**
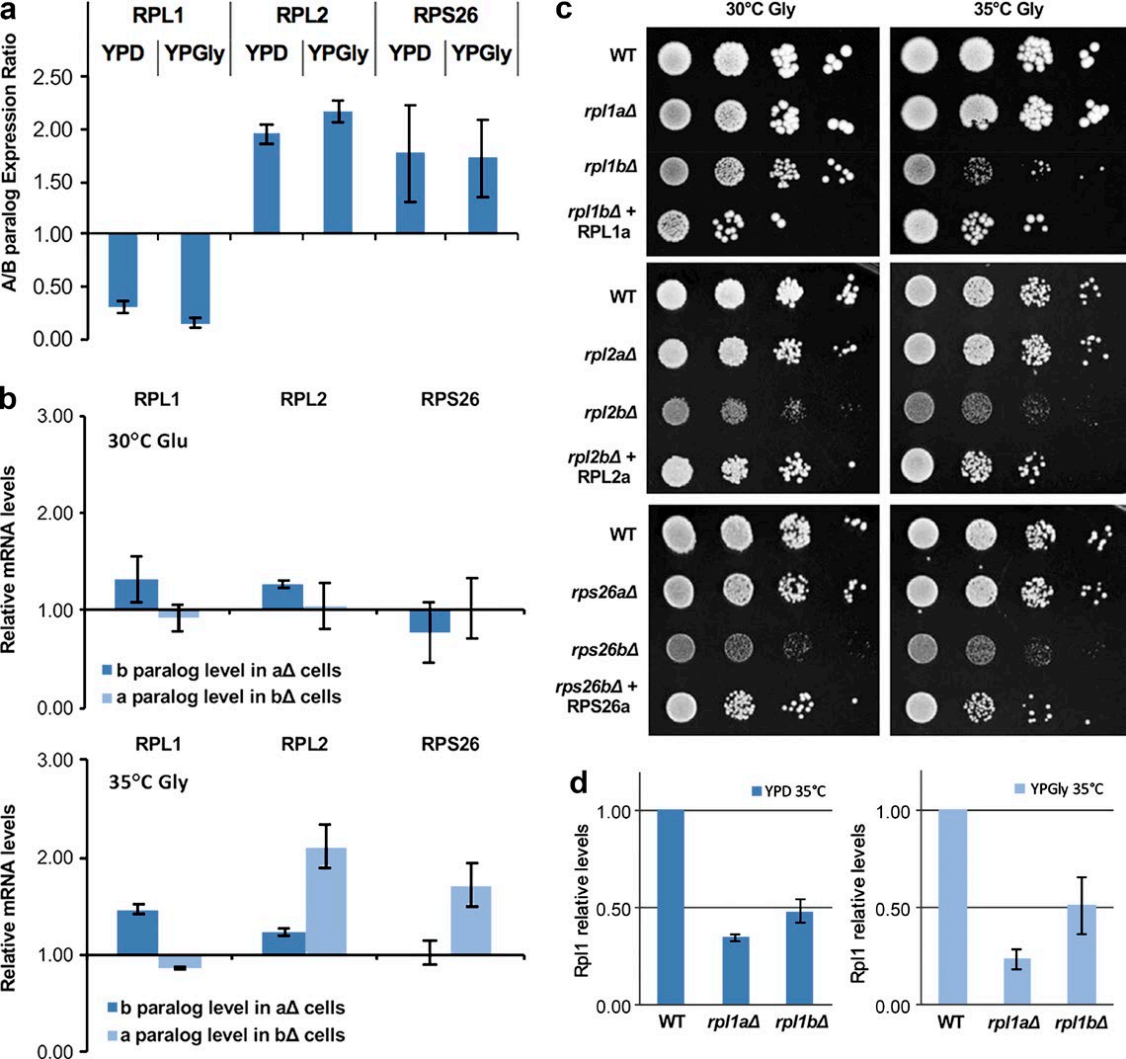
**RP paralog-specific phenotypes are not accompanied by changes in gene expression of the other paralog nor suppressed by its overexpression.** (a) RP paralog gene expression ratio does not change on fermentable versus nonfermentable carbon sources. WT cells were grown to mid–log phase on glucose (YPD) or glycerol (Gly; YPGly) at 35°C before RNA extraction and analysis by qRT-PCR using specific primers for each paralog. qRT-PCR experiments were performed in triplicate from three biological repeats. (b) Deletion of one RP paralog does not change expression of its cognate partner. RP deletion mutants were grown and analyzed by qRT-PCR as in panel a. Experiments were performed in triplicate from three biological repeats. mRNA levels are shown relative to WT expression. (c) Paralog overexpression only partially substitutes for the loss of its cognate partner. WT, RP deletion, and RP deletion strains overexpressing their corresponding *a* paralog from a single-copy plasmid were plated and grown on YPD or YPGly at 30°C and 35°C (Fig. S2). (d) Rpl1 protein levels decrease in both *rpl1aΔ* and *rpl1bΔ* mutants. WT, *rpl1aΔ*, and *rpl1bΔ* cells were grown to mid–log phase on YPD or YPGly at 35°C and processed for Western analysis. Rpl1 protein from each sample was quantified using GelQuantNet and normalized to total protein in each lane as detected by Ponceau staining (Fig. S2 c). No significant difference (P > 0.05) was detected between the Rpl1 levels in *rpl1aΔ* and *rpl1bΔ* cells. Western blotting and analysis was performed in triplicate from three biological repeats. Error bars represent SEM.

Both the HTML and PDF versions of the article have been corrected. This error appears only in PDF versions downloaded on or before January 31, 2018. Rockefeller University Press apologizes for this regrettable error.

